# Oral health-related quality of life and oral hygiene status among special need school students in amhara region, Ethiopia

**DOI:** 10.1186/s12955-023-02110-4

**Published:** 2023-03-20

**Authors:** Amare Teshome Tefera, Biruk Girma, Aynishet Adane, Abebe Muche, Tadesse Awoke Ayele, Kefyalew Ayalew Getahun, Zelallem Aniley, Semira Ali, Simegnew Handebo

**Affiliations:** 1 Department of Dentistry, School of Medicine, College of Medicine and Health Sciences, University of Gondar, Gondar, Ethiopia; 2 Department of Internal Medicine, School of Medicine, College of Medicine and Health Sciences, University of Gondar, Gondar, Ethiopia; 3 Department of Anatomy, School of Medicine, College of Medicine and Health Sciences, University of Gondar, Gondar, Ethiopia; 4 Department of Epidemiology and Biostatistics, Institute of public health, College of Medicine and Health Sciences, University of Gondar, Gondar, Ethiopia; 5 Department of Pharmacology, School of Pharmacy, College of Medicine and Health Sciences, University of Gondar, Gondar, Ethiopia; 6 Department of Special Needs and Inclusive Education, College of Education, University of Gondar, Gondar, Ethiopia; 7 School of Public Health, St. Paul’s Hospital Millennium Medical College, P.O.Box- 1271, Addis Ababa, Ethiopia

**Keywords:** Oral hygiene status, OHRQoL, Special needs, Disabilities, Students, Ethiopia

## Abstract

**Background:**

Oral conditions remain a substantial population health challenge worldwide. Poor oral health affects the quality of life as a result of pain or discomfort, tooth loss, impaired oral functioning, disfigurement, missing school time, loss of work hours, and sometimes even death. This study assessed the magnitude of Oral Health-Related Quality of Life (OHRQoL) and oral hygiene status and associated factors among special needs school students in the Amhara region.

**Methods:**

An institution-based cross-sectional study was conducted from November 2020 to April 2021 in the Amhara Region, Ethiopia. A total of 443 randomly selected special needs students were included. A structured pretested interview-administered questionnaire was used for data collection. Bivariable and multivariable ordinal logistic regression models were fitted to identify the factors associated with oral hygiene status. The statistical significance of differences in mean OHIP-14 scores was assessed using the Kruskal-Wallis equality-of-populations rank and Wilcoxon rank-sum tests. Variables with a p-value less than 0.05 were considered statistically significant.

**Results:**

Almost half 46.6% (95% CI: 42.1%, 51.4%) of the study participant had poor oral hygiene status. The median OHIP-14 score was 16 with an interquartile range from 14 to 20. The highest score was for functional limitation (mean: 1.45 (SD ± 0.70)) and the lowest score was for psychological disability (mean: 1.08 (SD ± 0.45)). Mother education, frequency of taking sugared foods, and the types of disabilities were significant predictors of the poor oral hygiene status of special needs students in the Amhara region. The students living in Dessie had higher OHIP-14 scores compared to those living in other places (Gondar, Bahir Dar, and Debre Markos). The students who never brush their teeth had lower OHIP-14 scores than those who brush sometime and once a day. Whereas, students affiliated with the orthodox religion had lower OHIP-14 scores compared to those affiliated with all other religions (Catholic, Muslim, and Protestant).

**Conclusion:**

A substantial amount of students with a disability had poor oral hygiene. The OHIP-14 scores indicated poor oral health-related quality of life. The study found that maternal education, frequency of taking sugared foods, and the types of disabilities were statistically significant factors associated with oral hygiene status.

## Background

Oral health is a key indicator of overall health, well-being, and quality of life [1], [2]. Globally, around 3.5 billion people are affected by oral disease, and the poor and disadvantaged groups are affected more [3]. The year of healthy life lost due to diseases or injuries to oral conditions increased by 8% from 16.9 to 18.3 million between 2015 and 2017 [4]. Poor oral health causes debilitating pain for millions around the world as well as rises the out-of-pocket financial burden on society. In addition to the toothache and reduced nutritional status, individuals with poor oral health status would have a psychological problems, and difficulty in performing daily activities in school and work settings [2], [5], [6] [7]. Moreover, individuals with poor oral health have an increased risk of medical conditions such as; diabetes, cardiovascular disease (CVD), and pneumonia [8–10].

Individuals living with disability have a compromised self-care practice due to physical and cognitive limitations and are at increased risk of dental caries and periodontal disease [11], [12]. Moreover, the frequent use of medicines having a higher sugar content, dependency on oral hygiene practice, reduced clearance of foods from the oral cavity, impaired salivary function, preference for carbohydrate-rich foods, a liquid or puréed diet, and oral aversions increase the oral health problems and conditions [12], [13].

The oral health status of children and young adults living with a disability is poor [14], [15]. Individuals living with disability have limited oral hygiene performance due to the presence of motor, sensory and intellectual disabilities [16], and these individuals may not understand and assume responsibility for or cooperate with preventive oral hygiene practices [17]. Moreover, many caregivers do not have the requisite knowledge or values to recognize the importance of oral hygiene and do not themselves practice appropriate oral hygiene or adhere to a proper diet [18].

Dental diseases and conditions negatively affect the quality of life of an individual with a disability and their family by causing difficulty eating, weight loss, and decreased nutritional status [19–21]. Oral health-related quality of life (OHRQoL) is a self-reported condition of oral health that assesses the functional, social, and psychological impacts of oral disease [22]. OHRQoL is an integral part of general health and well-being [23] and corresponds to the impact of oral diseases on an individual’s daily functioning and well-being [24], [25] [26]. The OHRQoL is evaluated using the oral health impact profile-14 (OHIP-14), which contains 14 questions and 7 domains (functional limitation, physical pain, psychological discomfort, physical disability, psychological disability, social disability, and handicap) [27]. Understanding the OHRQoL and oral health status of students living with disability has paramount importance in the design of oral health interventions and strategies among the disabled population.

To the best of our knowledge, there is no study on the OHRQoL and oral health status of Students living with disability in Ethiopia. Hence, this study aimed to assess the OHRQoL & oral hygiene status of students living with disability in the Amhara region, Ethiopia.

## Methods

### Study area

This institution-based cross-sectional study was conducted from November 2020 to April 2021 in special needs schools in the Amhara region, Ethiopia. Amhara region is found in the northern part of Ethiopia and Bahir-dar is the capital city of the region. Eight special needs schools were included in the study. In Ethiopia, only 4% of children with disabilities are currently attending school [28]. Children with only physical disabilities enrolled in ordinary schools, while special needs schools provide education for children with other types of disability as well as those who have both physical and other types of disability.

**Fig. 1:**
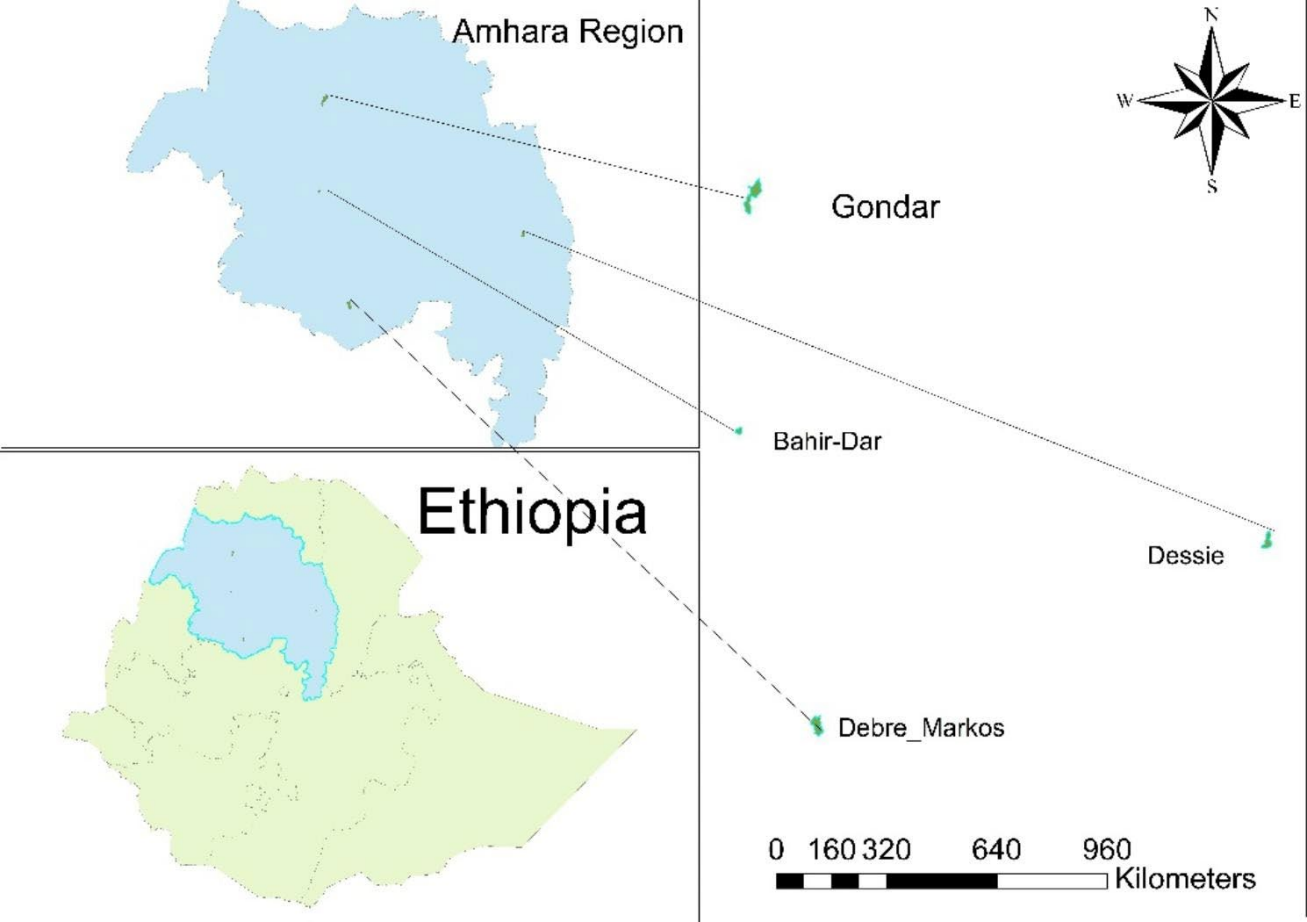
The study area. (source: Source for shape file-OpenAfrica)

### Study population

The study population were students in special needs schools living with hearing, visual, physical, and mental disabilities in the Amhara region. Students living with disability and attending special needs education in the region were included in the study. Students with uncooperative behavior, absent during the data collection period, and unable to provide data were excluded from the study. However, we did not excluded any student from the study.

### Sample size and sampling procedure

A single population proportion formula was used to determine the sample size, that was done for another study [29] considering a prevalence of 50% (no-previous study in the country), a 5% margin of error (d), 95% confidence level, and 15% non-response rate. Accordingly, the final sample size was 443 students living with a disability. To recruit study participants, a simple random sampling technique using a computer random number generator was used.

### Study variables

Oral hygiene status and oral health-related quality of life (OHRQoL) were the dependent variables. The independent variables were; sociodemographic characteristics (age, sex, religion, grade level, family educational status, and monthly income), tooth brushing, carbohydrates intake, self-reported dental health problems, treatment-seeking behavior, disability (types and duration).

### Operational definition

#### Oral hygiene status

The oral hygiene status was recorded based on a simplified oral hygiene index, OHI-S, and recorded as follows: 0–1.2, Good; 1.3–3.0, Fair; and 3.1-6.0, Poor [30].

### OHRQoL

The OHRQoL of the study participants was assessed using the oral health impact profile (OHIP-14), which measures the frequency of oral impacts on everyday life within the past year [27]. It contains 14 questions and 7 domains (functional limitation, physical pain, psychological discomfort, physical disability, psychological disability, social disability, and handicap). Responses were provided using 5-point ordinal scales (never = 0, hardly ever = 1, occasionally = 2, fairly often = 3 and very often = 4). Domain scores were calculated by adding the responses to the two corresponding items (range: 0 to 8) and the total score by adding the responses to all 14 items (range: 0 to 56). Higher scores indicated worse OHRQoL [31]. Summary OHIP-14 scores were calculated by summing ordinal values for 14 items. Higher OHIP-14 scores indicate worse and lower scores indicate a better oral health-related quality of life.

#### Hearing impairment

The term hearing impairment refers to students who had either complete or partial hearing problems.

#### Visual impairment

Visual impairment refers to an impairment in vision that, even with correction, adversely affects a child’s educational performance. The term includes both partial sight and blindness.

### Data collection procedure

Data were collected using a pretested structured interview-administered questionnaire that was adapted from the WHO oral health survey, OHIP-14, and other literature [27], [32]. In the very beginning, the tool was prepared in English and then translated into the local Amharic language. To check the consistency of the questionnaire, the Amharic version was translated back to English. A pretest was done on 23 students (5%) at Injibara, which is not selected for the main study. Based on the input from the pretest, unclear questions were modified, wording in the questionnaire was improved and the order of the questions was rearranged. Dentists and special needs teachers were involved in the data collection process.

The intra-oral examination was done by four dentists at the schools using normal light, a tongue depressor, mouth mirror, periodontal probe, and dental explorers. The DMFT index (decayed, missed, and filled teeth) and oral hygiene status (based on simplified oral hygiene index, OHI-S) data were recorded.

A five days training was given to the data collectors and were calibrated on the data collection procedure, content of the instrument, and ethical considerations during data collection. Throughout the data collection procedures, COVID-19-related safety precautions were undertaken. Each returned questionnaire was reviewed for completeness and consistency on daily basis.

### Data analysis

The collected data were entered into Epi-Data (version 4.6) and exported into STATA (version 14) for analysis. Descriptive analyses such as median, mean, proportion, standard deviation, and frequency were computed. The reliability test of OHIP-14 items for Cronbach’s alpha was 0.81. The normality of OHRQoL was assessed with its kurtosis and skewness values. Since the score of all domains didn’t follow the normal distribution, we employed Spearman’s correlation analysis to assess the relationship among OHRQoL dimensions. In addition, the statistical significance of differences in mean OHIP-14 scores was assessed using the Kruskal-Wallis equality-of-populations rank test and Wilcoxon rank-sum (Mann-Whitney) tests. The association between oral hygiene status and socio-demographic independent variables was assessed using Chi-square tests. Bivariable and a multivariable ordinal logistic regression model was fitted to identify the factors associated with oral hygiene status. Those variables with a p-value of less than 0.25 in the bivariable ordinal logistic regression model were fitted in the multivariable model. Variables with a p-value of < 0.05 at a 95% confidence interval were considered statistically significant.

## Results

### Socio-demographic factors

Four hundred forty-three students participated in the study with a response rate of 100%. This was attributed to the repeated visit that the data collectors to each special need schools and excluded students who were uncooperative and did not attend the whole semester, and also the schools were dedicated only for this disabled individual that has its own day care center. The mean age of the participants was 15.84 ± 3.88, and the majority (64.3%) of them were in the age range of 13 to 18 years. More than half (53.5%) of the participants were males. About one-third (33.6%) of the participants were hearing impaired.

The chi-square test of association was used to determine if there was any association between oral hygiene status and socio-demographic variables. Accordingly, the finding revealed that age (P = 0.001), religion (P = 0.001), mother educational status (P = 0.003), disability type (P = 0.001), and monthly family income (P = 0.006) were significantly associated with the oral hygiene status of students with disabilities (Table 1).

**Table 1:** Cross-tabulation and chi-square test of oral hygiene status with the characteristics of special need school students in Amhara regional state, Ethiopia, 2021 (n = 443)

Variables		Oral hygiene status			P-value
		**Good n (%)**	**Fair n (%)**	**Poor n (%)**	
Sex	Male	41 (17.3)	88 (37.1)	108 (45.6)	0.181
	Female	46 (22.3)	61 (29.6)	99 (48.1)	
Age	7–12	29 (38.6)	23 (30.7)	23 (30.7)	0.001
	13–18	48 (16.8)	98 (34.4)	139 (48.8)	
	> 18	10 (12.1)	28 (33.7)	45 (54.2)	
Location of the participants	Gondar	23 (25.0)	29 (31.5)	40 (43.5)	0.182
	Bahir Dar	14 (9.7)	60 (41.7)	70 (48.6)	
	Debre Markos	12 (9.0)	40 (30.1)	81 (60.9)	
	Dessie	38 (51.4)	20 (27.0)	16 (21.6)	
Grade Level	1–4	45 (19.1)	78 (33.0)	113 (47.9)	0.150
	5–8	36 (24.2)	52 (34.9)	61 (40.9)	
	9–12	6 (10.3)	19 (32.8)	33 (56.9)	
Religion	Orthodox	54 (17.5)	107 (34.6)	148 (47.9)	0.001
	Catholic	7 (11.1)	17 (27.0)	39 (61.9)	
	Muslim	25 (40.3)	20 (32.3)	17 (27.4)	
	Protestant	1 (11.1)	5 (55.6)	3 (33.3)	
Mother educational status	No education	40 (15.6)	79 (30.7)	138 (53.7)	0.003
	Able to read and write	26 (23.0)	42 (37.2)	45 (39.8)	
	Formal education	17 (32.7)	20 (38.5)	15 (28.8)	
Father educational status	No education	37 (18.8)	66 (33.5)	94 (47.7)	0.124
	Able to read and write	24 (16.9)	47 (33.1)	71 (50.0)	
	Formal education	23 (29.1)	29 (36.7)	27 (34.2)	
Disability types	Visual impairment	21 (16.2)	52 (40.0)	57 (43.8)	0.001
	Hearing impairment	54 (36.3)	48 (32.2)	47 (31.5)	
	Mental problem	6 (4.4)	40 (29.2)	91 (66.4)	
	Other*	6 (22.2)	9 (33.3)	12 (44.4)	
Monthly family income	Less than 1000	141 (51.3))	84(30.5)	50 (18.2))	0.006
	1000–2500	22 (32.4)	30 (44.1)	16 (23.5)	
	Above 2500	15 (31.91)	16 (34.04)	16 (34.04)	
Tooth brushing	Never	17 (16.0)	42 (39.6)	47 (44.3)	0.151
	Sometimes	32 (17.3)	56 (30.3)	97 (52.4)	
	Once a day	30 (25.6)	42 (35.9)	45 (38.5)	
	Twice a day	8 (22.9)	9 (25.7)	18 (51.4)	
Self-reported dental health problem	No	43 (18.0)	89 (37.2)	107 (44.8)	0.207
	Yes	44 (21.6)	60 (29.4)	100 (49.0)	
Dental caries	Yes	25 (13.6)	54 (29.3)	105 (57.1)	0.001
	No	62 (23.9)	95 (36.7)	102 (39.4)	
Sought dental care	No	10 (12.4)	24 (29.6)	47 (58.0)	0.022
	Yes	34 (27.6)	36 (29.3)	53 (43.1)	
Frequency of carbohydrate foods	Never	9 (21.9)	13 (31.7)	19 (46.3)	0.077
	Sometimes	39 (19.7)	62 (31.3)	97 (49.0)	
	Once a day	16 (23.2)	33 (47.8)	20 (29.0)	
	Twice a day	18 (17.8)	27 (26.7)	56 (55.5)	
	Three and more times a day	5 (14.7)	14 (41.2)	15 (44.1)	

_* Multiple disabilities_

### Oral health-related quality of life

The mean OHIP-14 score was 17.97 (SD ± 5.31) with a range of 4 to 48. The highest score was for functional limitation (mean: 1.45 (SD ± 0.70) and the lowest score was for psychological disability (mean: 1.08 (SD ± 0.45) **(**Table 2**).**

**Table 2:** Descriptive summary results of oral health-related quality of life (OHIP-14) dimensions of special need school students in Amhara regional state, Ethiopia, 2021 (n = 443)

OHIP dimensions	Number of items	Mean (SD)	Median (IQR)	Skewness	Reliability
Functional limitation	2	1.45 (0.70)	1 (1)	0.91	0.15
Physical pain	2	1.40 (0.64)	1 (1)	1.61	0.72
Psychological discomfort	2	1.44 (0.65)	1 (1)	0.87	0.14
Physical disability	2	1.18 (0.48)	1 (0)	2.68	0.78
Psychological disability	2	1.08 (0.45)	1 (0)	1.87	0.75
Social disability	2	1.22 (0.51)	1 (0.5)	2.35	0.51
Handicap	2	1.21 (0.51)	1 (0.5)	2.08	0.69
Physical domain	6	1.34 (0.46)	1.17 (0.5)	1.66	0.66
Psychological domain	4	1.26 (0.45)	1 (0.5)	1.28	0.49
Social Domain	4	1.28 (0.48)	1 (0.25)	2.26	0.80
OHIP-14*	14	17.97 (5.31)	16 (6)	2.04	0.81

*Un-standardized score, IQR = interquartile range, OHIP = oral health-related quality of life, SD = standard deviation

There was a significant positive correlation among the OHRQoL dimensions except between functional limitation and psychological discomfort. Social disability showed the strongest correlation to the handicap as compared to all other dimensions (Table 3).

**Table 3:** Correlation among oral health-related quality of life (OHIP-14) dimensions

Constructs	1	2	3	4	5	6	7
1. Functional limitation	1.00						
2. Physical pain	0.17*	1.00					
3. Psychological discomfort	-0.07	0.36*	1.00				
4. Physical disability	0.26*	0.60*	0.34*	1.00			
5. Psychological disability	0.20*	0.38*	0.33*	0.49*	1.00		
6. Social disability	0.45*	0.53*	0.27*	0.51*	0.35*	1.00	
7. Handicap	0.41*	0.59*	0.28*	0.57*	0.28*	0.75*	1.00

* significant correlation coefficient at p-value < 0.05

### Comparison of OHIP-14 domain by study participant’s characteristics

The Table 4 presents the distribution of OHIP-14 domains by participants’ characteristics. The psychological domain score of students aged greater than 18 years was significantly higher compared to their younger counterparts (median = 1.50 (IQR 0.8), P < 0.001). The physical domain (median = 1.50 (IQR 0.7)), social domain (median = 1.75 (IQR 1.3)), and Overall OHIP-14 (median = 1.50 (IQR 0.5)) scores were significantly higher in students living in Dessie compared to those living in other places (P < 0.001). Yet, students from Debre Markos had higher psychological domain scores compared to those living in other places (median = 1.50 (IQR 0.8), P < 0.001). The physical score was significantly higher in students with primary education compared to those with upper education (median = 1.30 (IQR 0.5), P = 0.0019). Students affiliated with the Orthodox religion had lower physical domain (median = 1.00 (IQR 0.5)), psychological domain (median = 1.00 (IQR 0.5)), social domain (median = 1.00 (IQR 0.0)), and Overall OHIP-14 (median = 1.14 (IQR 0.2)) scores compared to those affiliated with other religions (P < 0.001). Hearing impaired students had higher physical (median = 1.33 (IQR 0.7)) and social domain (median = 1.00 (IQR 0.8)) scores (P < 0.001). On the other hand, students with other classifications of impairment had higher psychological (median = 1.50 (IQR 0.8), P < 0.001) compared to students with hearing and mental impairment. The physical domain (median = 1.17 (IQR 0.3), P = 0.0083) and social domain (median = 1.00 (IQR 0.0), P = 0.0023) scores of students who never brush their tooth was lower than those who brush once a day. The psychological domain scores of students who never brush their tooth was lower than those who brush sometime (median = 1.00 (IQR 0.3) p = 0.0012). The overall OHIP-14 scores of students who never brush their tooth was lower than those who brush sometime and once a day (P < 0.001). The psychological (median = 1.00 (IQR 0.5)) and social domain (median = 1.00 (IQR 0.8) scores in the students with good oral hygiene status were significantly higher than those with poor and others, respectively (P = 0.0237 and P = 0.0043, respectively). Dental caries, self-reported dental health problems, and sex differences did not significantly affect OHIP-14 scores. (Table 4).

**Table 4:** Distribution of OHIP dimensions with study participant’s characteristics

Explanatory variable		Physical dimension	Psychological dimension	Social dimension	Overall OHIP-14
Age	7–12	1.33 (IQR 0.5)	1.00 (IQR 0.3)	1.00 (IQR 0.5)	1.14 (IQR 0.4)
	13–18	1.17 (IQR 0.5)	1.00 (IQR 0.5)	1.00 (IQR 0.3)	1.14 (IQR 0.4)
	> 18	1.17 (IQR 0.5)	1.50 (IQR 0.8)	1.00 (IQR 0.3)	1.21 (IQR 0.4)
	p-value ^#^	0.601	< 0.001	0.451	0.207
Location of the participants	Gondar	1.17 (IQR 0.7)	1.25(IQR 0.8)	1.00 (IQR 0.4)	1.21 (IQR 0.6)
	Bahir Dar	1.00 (IQR 0.3)	1.00 (IQR 0.0)	1.00 (IQR 0.0)	1.00 (IQR 0.2)
	Debre Markos	1.17 (IQR 0.5)	1.50 (IQR 0.8)	1.00 (IQR 0.3)	1.14 (IQR 0.4)
	Dessie	1.50 (IQR 0.7)	1.25 (IQR 1.3)	1.75 (IQR 1.3)	1.50 (IQR 0.5)
	p-value ^#^	< 0.001	< 0.001	< 0.001	< 0.001
Grade Level	1–4	1.30 (IQR 0.5)	1.00 (IQR 0.5)	1.00 (IQR 0.3)	1.21 (IQR 0.4)
	5–8	1.00 (IQR 0.5)	1.25 (IQR 0.8)	1.00 (IQR 0.5)	1.21 (IQR 0.5)
	9–12	1.00 (IQR 0.3)	1.25 (IQR 0.8)	1.00 (IQR 0.3)	1.14 (IQR 0.4)
	p-value ^#^	0.0019	0.414	0.414	0.357
Religion	Orthodox	1.00 (IQR 0.5)	1.00 (IQR 0.5)	1.00 (IQR 0.0)	1.14 (IQR 0.2)
	Catholic	1.33 (IQR 0.8)	1.25 (IQR 0.5)	1.00 (IQR 0.5)	1.21 (IQR 0.4)
	Muslim	1.50 (IQR 0.8)	1.25 (IQR 0.8)	1.25 (IQR 1.0)	1.36 (IQR 0.6)
	Protestant	1.33 (IQR 0.5)	1.75 (IQR 0.8)	1.00 (IQR 0.3)	1.21 (IQR 0.2)
	p-value^#^	< 0.001	0.001	< 0.001	< 0.001
Disability types	Visual impaired	1.00 (IQR 0.3)	1.25 (IQR 0.8)	1.00 (IQR 0.3)	1.14 (IQR 0.4)
	Hearing impaired	1.33 (IQR 0.7)	1.00 (IQR 0.5)	1.00 (IQR 0.8)	1.21 (IQR 0.6)
	Mental problem	1.33 (IQR 0.5)	1.00 (IQR 0.3)	1.00 (IQR 0.0)	1.14 (IQR 0.2)
	Others	1.16 (IQR 1.0)	1.50 (IQR 0.8)	1.00 (IQR 0.5)	1.29 (IQR 0.7)
	p-value ^#^	< 0.001	< 0.001	< 0.001	0.027
Frequency of tooth brushing	Never	1.17 (IQR 0.3)	1.00 (IQR 0.3)	1.00 (IQR 0.0)	1.07 (IQR 0.2)
	Sometimes	1.17 (IQR 0.5)	1.25 (IQR 0.8)	1.00 (IQR 0.3)	1.21 (IQR 0.4)
	Once a day	1.33 (IQR 0.7)	1.00 (IQR 0.5)	1.00 (IQR 0.8)	1.21 (IQR 0.6)
	Twice a day	1.00 (IQR 0.5)	1.25 (IQR 0.5)	1.00 (IQR 0.3)	1.14 (IQR 0.4)
	p-value ^#^	0.0083	0.0012	0.0023	< 0.001
Oral hygiene status	Good	1.33 (IQR 0.7)	1.00 (IQR 0.5)	1.00 (IQR 0.8)	1.21 (IQR 0.6)
	Fair	1.17 (IQR 0.5)	1.00 (IQR 0.5)	1.00 (IQR 0.3)	1.14 (IQR 0.4)
	Poor	1.17 (IQR 0.5)	1.00 (IQR 0.5)	1.00 (IQR 0.3)	1.14 (IQR 0.4)
	p-value ^#^	0.134	0.0237	0.0043	0.222
Sex	Male	1.17 (IQR 0.5)	1.00 (IQR 0.5)	1.00 (IQR 0.3)	1.14 (IQR 0.4)
	Female	1.17 (IQR 0.5)	1.00 (IQR 0.5)	1.00 (IQR 0.3)	1.14 (IQR 0.4)
	p-value ^&^	0.854	0.305	0.329	0.870
Self-reported dental health problem	No	1.50 (IQR 0.7)	1.25 (IQR 0.8)	1.00 (IQR 0.5)	1.29 (IQR 0.5)
	Yes	1.50 (IQR 0.7)	1.25 (IQR 0.8)	1.25 (IQR 0.8)	1.43 (IQR 0.5)
	p-value^&^	0.241	0.401	0.273	0.243
Dental caries	Yes	1.33 (IQR 0.7)	1.25 (IQR 0.8)	1.00 (IQR 0.5)	1.14 (IQR 0.2)
	No	1.00 (IQR 0.5)	1.00 (IQR 0.5)	1.00 (IQR 0.0)	1.21 (IQR 0.5)
	p-value^&^	0.641	0.401	0.273	0.243

#: Kruskal-Wallis equality-of-populations rank test, &: wilcoxon rank-sum (Mann-Whitney) test

### Oral hygiene status

About half (46.6%, 95% CI: 42.1%, 51.4%) of the study participants had poor oral hygiene status. Only 19.6% of special needs students had good oral hygiene status.

### Factors associated with oral hygiene status

After assumptions of the proportional odds model were assessed eleven variables were identified as the candidates for multivariable ordinal logistic regression. Mother education, frequency of taking carbohydrate foods, and the types of disabilities were statistically significant factors associated with the oral hygiene status of special needs students in the Amhara region. Given the other variables in the model constant, for students whose mothers were able to read and write (AOR = 3.85, 95% CI: (1.55, 9.53)) and had formal education (AOR = 4.56, 95% CI: (1.25, 16.58)), the odds of good oral hygiene versus the combined fair and poor oral hygiene were 3.85 and 4.56 times higher than for students whose mothers had no education, respectively. Likewise, the odds of the combined good and fair oral hygiene versus poor oral hygiene were 3.85 and 4.56 times higher for students whose mothers were able to read and write and had formal education compared to students whose mothers had no education, respectively, given the other variables were held constant in the model.

For students taking sugary foods ≥ 3 times a day, the odds of good oral hygiene versus the combined fair and poor oral hygiene were 6.63 times higher than for students who never took sugary foods, given the other variables are held constant. Likewise, the odds of the combined categories of good and fair oral hygiene versus poor oral hygiene are 6.63 times higher for students who took sugary food three and more times a day compared to students who never took sugary foods, given the other variables are held constant in the model (AOR = 6.63, 95% CI: (1.10, 39.79)). Keeping other variables in the model constant, the likelihood of mentally impaired students having good oral hygiene as opposed to fair or poor oral hygiene was 0.15 times lower than visually impaired students (AOR = 0.15, 95% CI: (0.05, 0.48)) (Table 5).

**Table 5:** Factors associated with oral hygiene status among special needs students in Amhara region, Ethiopia, 2021 (n = 443)

Independent variables		COR (95% CI)	AOR (95% CI)	P-value
Age of the participant^&^		0.91 (0.87, 0.95)	0.92 (0.79, 1.06)	0.264
Grade	1–4 grade	1	1	
	5–8 grade	1.34 (0.91, 1.96)	1.17 (0.51, 2.66)	0.713
	9–12 grade	0.65 (0.38, 1.14)	0.57 (0.15, 2.08)	0.391
Religion	Orthodox	1	1	
	Catholic	0.57 (0.33, 0.98)	1.77 (0.61, 5.16)	0.293
	Muslim	2.84 (1.69, 4.77)	1.55 (0.68, 3.53)	0.293
	Protestant	1.31 (0.41, 4.15)	0.39 (0.04, 3.72)	0.416
Mother educational status	No education	1	1	
	Able to read and write	1.71 (1.13, 2.59)	3.85 (1.55, 9.53)	0.004**
	Formal education	2.77 (1.59, 4.83)	4.56 (1.25, 16.58)	0.021*
Father educational status	No education	1	1	
	Able to read and write	0.90 (0.60, 1.36)	0.69 (0.30, 1.63)	0.404
	Formal education	1.78 (1.09, 2.88)	1.63 (0.54, 4.94)	0.388
Family income	Less than 1000 ETB	1	1	
	1000-2500ETB	1.83 (1.13, 2.97)	1.32 (0.57, 3.07)	0.524
	Greater than 2500 ETB	2.33 (1.31, 4.17)	0.89 (0.25, 3.12)	0.858
Frequency of carbohydrate foods	Never	1	1	
	Sometimes	0.89 (0.47, 1.68)	2.23 (0.61, 8.21)	0.227
	Once a day	1.59 (0.78, 3.25)	2.23 (0.51, 9.76)	0.287
	Twice a day	0.70 (0.35, 1.40)	1.44 (0.35, 5.91)	0.616
	Three and more times a day	0.93 (0.40, 2.16)	6.63 (1.10, 39.79)	0.039*
Frequency of tooth brushing	Never	1	1	
	Sometimes	0.81 (0.52, 1.26)	0.60 (0.23, 1.53)	0.284
	Once a day	1.41 (0.87, 2.29)	0.68 (0.23, 1.99)	0.480
	Twice a day	0.93 (0.44, 1.92)	0.33 (0.06, 1.68)	0.181
Disability types	Visually impaired	1	1	
	Hearing impaired	2.17 (1.39, 3.38)	0 0.88 (0.35, 2.19)	0.782
	Mental impaired	0.39 (0.25, 0.63)	0.15 (0.05, 0.48)	0.001**
	Others^#^	1.11 (0.51, 2.42)	1.15 (0.22, 6.03)	0.872
Sought dental care	No	1	1	
	Yes	2.02 (1.18, 3.46)	1.60 (0.78, 3.30)	0.201
Years lived with disability^&^		0.92 (0.86, 0.96)	1.05 (0.92, 1.19)	0.485

_Note: # multiple disabilities, & continuous variable, * significant at p value < 0.05, ** Significant at p−value <0.01 COR: Crudes Odds Ratio, AOR: Adjusted Odds Ratio_

## Discussion

This study assessed the oral hygiene status and oral health-related quality of life of special needs school students in the Amhara Region, Ethiopia. Nearly half of the study participants had poor oral hygiene status. The mean OHIP-14 score was 17.97 (SD ± 5.31) with range of 4 to 48. The highest score was for functional limitation and the lowest score was for psychological disability.

Children living with disability has poor oral hygiene status than non-disabled children [33]. Consistent with such findings, the current study found that half of the study participants had poor oral hygiene status. Our finding is higher than a previous study done in Nigeria, Lagos where 22.2% of special needs individuals had poor oral hygiene status [17]. Similarly, a study in Yemen also reported a high prevalence of caries and poor oral hygiene status among children with special needs [14]. On the other hand, a study done among Arabian children with special health care needs found that 64.8% of the sample have good oral hygiene [34]. These variations might be due to the difference in socio-demographic factors among the study populations. Oral hygiene practice can be challenging for uncooperative children and those with severe disabilities. In children with impairments, poor oral hygiene has been identified as the most important factor of caries risk [14].

In the current study, the results of the OHIP-14 questionnaire indicated a moderate oral health-related quality of life among students living with a disability. The mean OHIP-14 score was 17.97 (SD ± 5.31). Similar findings have been reported for elderly people in Nigeria [35], Iran [36], and Iraq [37]. Another study in Brazil reported that 68.7% of children with oral hygiene problems had a negative oral health quality of life [20]. This could be explained by the differences in research participants and the presence of severe oral symptoms among individuals who visit dental clinics, among which oral hygiene problems have a greater influence on the quality of life. Our finding showed that the highest score of OHRQoL was observed for functional limitation (mean 1.45 (SD ± 0.70). It indicates that the subjects had difficulties in speaking, hygiene, occupational, and eating.Studies on the older population observed that the most commonly affected domains of the OHIP-14 questionnaires were the physical pain and psychological discomfort [38], [39]. Other studies also observed the highest score in the domain of psychological discomfort [36], [40].

Our study also found that students with disability living in Dessie had significantly higher OHIP-14 scores. This could be attributed to socio-cultural difference in the study settings. Besides students with disability affiliated with the Orthodox religion and never brush their tooth had significantly lower OHIP-14 scores. This is due to tooth brushing enable people to keep their mouth clean, remove food and plaque, and prevent the leading complications, which ultimately associated with improved OHRQoL. A review reported a difference in four dimensions of OHRQoL with the place of residence, in which urban adolescents had better scores than the rural ones [41]. A study done in Iraq found a statistically significant correlation between OHRQoL and tooth brushing frequency [37]. A review study reported that tooth brushing once per day is sufficient to maintain oral health and prevent caries and periodontal diseases. However, most people are not able to achieve sufficient plaque removal by performing oral hygiene measures at home. Therefore, tooth brushing twice daily is recommended to improve plaque control. This rule is followed by most people for their oral hygiene and has shown to be effective in maintaining oral hygiene and related quality of life [42]. In addition, the American Academy of Pediatric Dentistry recommended that tooth brushing should be performed for children twice daily [43]. In our study, there was no significant difference in OHIP-14 scores by gender, age group, presence of dental caries, and self-reported dental health problem.

Our study revealed that the oral hygiene status of special needs students was associated with maternal educational level, frequency of carbohydrate intake, and the types of disabilities. Students from mothers who can read and write and had formal education had good oral hygiene than their counterparts. Parents play a critical role in children’s oral hygiene status by controlling their feeding habits, oral hygiene care, and making services available to them [44]. In support of this, a study done in Wuhan reported that children who have better-educated parents tend to perform better oral hygiene practices, specifically, they will be more likely brush teeth more often, visit the dentist more frequently, and have regular dental check-up [45].

Carbohydrate foods play a major role in the development of dental caries. Bacteria within the plaque use the sugar as energy and release acid as a waste product, which gradually dissolves the enamel part of the tooth [46]. The World Health Organization (WHO) strongly advises people to limit their intake of free sugars throughout their lives. The intake of free sugars should be limited to 10% of total energy intake for both children and adults [47], [48]. Frequent consumption of carbohydrates in the form of simple sugars increases the risk of dental caries [49]. Contrary to this, our study found that children who took carbohydrate food three or more times a day were more likely to have good oral hygiene. Our result is contrary to the findings reported in Yemen where a higher prevalence of dental caries was found among those who frequently took carbohydrate foods [14]. This may be due to appropriate tooth brushing practice that reduced the impact of frequent carbohydrate foods intake [50]. This means the removal of biofilm on tooth surfaces, fundamental to the development of dental caries, through adequate oral hygiene may contribute to the prevention and control of dental health problems [51]. Our study also found that mentally impaired students had a lower prevalence of good oral hygiene status than visually impaired students. This could be associated with behavior and difficulties for parents/caregivers of mental disabilities to provide/support oral hygiene practices.

Similarly, a study conducted in Yemen found that students with mental problems had the highest rate of dental caries, while the blind had the lowest rate. This may be due to decreased tooth brushing habits, increased thirst, and long-term medication intake among mentally impaired children [17]. But a study done elsewhere reported that children with autism had good oral hygiene [52].

### Strength and limitation

This study is the first in assessing oral hygiene status and OHRQoL among students living with disability in Ethiopia. The finding of this study needs to be interpreted considering some limitations. Since we have used special needs language translators, some of the students did not express their problems confidentially. The study only included study participants from the special needs schools, children with disability who did not attend special education were excluded. Even among those who attended special needs schools, those study participants who were unable to express their problems and respond to the interview questions due to disability were excluded from the study. The causal relationship between the independent and dependent variables could not be established in advance due to the cross-sectional nature of the study design. Finally, due to a COVID-19 infection, some of the students withdrew from their academies and were not addressed during the data collection time.

## Conclusion

Overall, special needs school students with a disability had poor oral hygiene status and associated OHRQoL. This condition requires the adoption of strong prevention and control strategies at the individual and collective levels. Dentists should also need to organize public awareness campaigns and advise parents, caregivers, and patients on how to maintain good dental health. The study found that maternal education, frequency of taking sugared foods, and the types of disabilities were statistically significant factors associated with oral hygiene status. The students living in Dessie had higher OHIP-14 scores. Whereas, students affiliated with the orthodox religion and never brush their tooth had higher OHIP-14 scores. The improvement of children with disability’s OHRQoL can be achieved by educating parents/caregivers about the importance of home-based preventive measures and obtaining their assistance in doing so. For the dentist, healthcare professional, and policymakers, the relationship between oral health issues and the quality of life was an essential indicator that revealed the importance of oral health and equal access to such care.

## Data Availability

All datasets related to this article will be available upon a reasonable request from the corresponding author (Simegnew Handebo, E-mail: simegnewh@gmail.com).

## Abbreviations

AOR: Adjusted Odds Ratio
CI: Confidence Interval
COVID: Coronavirus Disease
CRPD: Convention on the Rights of Persons with Disabilities
COR: Crude Odds Ratio
IQR: Inter Quartile Range
OHIP-14: Oral Health Impact Profile-14
OHRQoL: Oral Health-Related Quality of Life
SD: Standard Deviation
WHO: World Health Organization
